# Refined localization of the *FAT1 *quantitative trait locus on pig chromosome 4 by marker-assisted backcrossing

**DOI:** 10.1186/1471-2156-7-17

**Published:** 2006-03-17

**Authors:** Frida Berg, Susanne Stern, Kjell Andersson, Leif Andersson, Maria Moller

**Affiliations:** 1 Department of Medical Biochemistry and Microbiology, Uppsala University, Uppsala, SE-751 24, Sweden; 2 Department of Animal Breeding and Genetics, Swedish University of Agricultural Science, Uppsala, SE-750 07, Sweden; 3 Experimental Medicine Unit, School of Medicine, University of Wales, Swansea, SA2 8PP, Wales, UK

## Abstract

**Background:**

A major QTL for fatness and growth, denoted *FAT1*, has previously been detected on pig chromosome 4q (SSC4q) using a Large White – wild boar intercross. Progeny that carried the wild boar allele at this locus had higher fat deposition, shorter length of carcass, and reduced growth. The position and the estimated effects of the *FAT1 *QTL for growth and fatness have been confirmed in a previous study. In order to narrow down the QTL interval we have traced the inheritance of the wild boar allele associated with high fat deposition through six additional backcross generations.

**Results:**

Progeny-testing was used to determine the QTL genotype for 10 backcross sires being heterozygous for different parts of the broad *FAT1 *region. The statistical analysis revealed that five of the sires were segregating at the QTL, two were negative while the data for three sires were inconclusive. We could confirm the QTL effects on fatness/meat content traits but not for the growth traits implying that growth and fatness are controlled by distinct QTLs on chromosome 4. Two of the segregating sires showed highly significant QTL effects that were as large as previously observed in the F_2 _generation. The estimates for the remaining three sires, which were all heterozygous for smaller fragments of the actual region, were markedly smaller. With the sample sizes used in the present study we cannot with great confidence determine whether these smaller effects in some sires are due to chance deviations, epistatic interactions or whether *FAT1 *is composed of two or more QTLs, each one with a smaller phenotypic effect. Under the assumption of a single locus, the critical region for *FAT1 *has been reduced to a 3.3 cM interval between the *RXRG *and *SDHC *loci.

**Conclusion:**

We have further characterized the *FAT1 *QTL on pig chromosome 4 and refined its map position considerably, from a QTL interval of 70 cM to a maximum region of 20 cM and a probable region as small as 3.3 cM. The flanking markers for the small region are *RXRG *and *SDHC *and the orthologous region of *FAT1 *in the human genome is located on HSA1q23.3 and harbors approximately 20 genes. Our strategy to further refine the map position of this major QTL will be i) to type new markers in our pigs that are recombinant in the QTL interval and ii) to perform Identity-By-Descent (IBD) mapping across breeds that have been strongly selected for lean growth.

## Background

We have previously reported a major quantitative trait locus (QTL), denoted *FAT1*, with large effects on fatness and growth located on SSC4q using a wild boar intercross [1,2]. Progeny that carried the wild pig chromosome 4 segment had higher fat deposition, shorter length of carcass, and reduced growth. QTL for fat deposition and growth located on pig chromosome 4 has also been found in other crosses e.g., Chinese Meishan vs. Large White [3,4], Iberian vs. Landrace [5,6] as well as in crosses of commercial populations [7,8]. Furthermore, a joint analysis comprising almost 3000 animals from seven different F2 crosses provided overwhelming statistical support for QTLs affecting fatness and growth on SSC4 [9]. The results from the different studies suggest that there most likely is more than one locus affecting body composition on this chromosome.

The position and the estimated effects of the *FAT1 *QTL for growth and fatness were confirmed in a backcross population of our wild boar pedigree [10]. Eighty-five offspring from two boars, one carrying a recombinant wild boar/Large White haplotype, were used for progeny testing. Both boars were found to be segregating for *FAT1 *and the interval could be determined to about 70 cM with the microsatellites *Sw871 *and *S0097 *as flanking markers. However, the presence of a second QTL proximal to *Sw871 *could not be excluded.

A recent comparative genome analysis revealed that *FAT1 *is located in a region orthologous to human chromosome 1q22-24 (HSA1q22-24) [11]. This region on HSA1q has previously been shown to harbor a locus for Type II diabetes identified in Pima Indians and Caucasian families [12,13] and a locus for familial combined hyperlipidemia [14]. The latter has been linked to the gene encoding upstream transcription factor 1 (*USF1*) [15].

In this study we have traced the inheritance of the wild boar QTL allele through marker-assisted backcrossing for an additional six generations in order to narrow down the *FAT1 *interval. For each backcross generation new boars, with a smaller and smaller portion of the wild pig derived segment of chromosome 4 were selected. These boars were then backcrossed to Large White sows and approximately 50 progeny from each recombinant were generated. We have also tested for the possible existence of a second QTL proximal of the *Sw871 *locus as indicated by Marklund *et al*. [10].

## Results

### Genotyping and marker development

The markers used for the QTL analyses are listed in Table 1. Two new microsatellites were isolated in this study, *S0832 *[GenBank: DQ218447] isolated from BAC RPCI44-310B8, which includes the *SDHC *gene, and *S0833 *[GenBank: DQ218446] isolated from BAC RPCI44-391C14, which includes the *PEA15 *gene. Both microsatellites are (GT)_n_-dinucleotide repeats. The observed size range for microsatellite *S0832 *was 243–258 bp; the two founder wild boars were homozygous for allele 243 while alleles 256 and 258 were most common among the Large White founders. For microsatellite *S0833 *the observed size range was between 152–177 bp; the two parental wild boars were homozygous for allele 156 and for the Large White the most common alleles observed were 152, 161, 163 and 167.

**Table 1:** Genetic markers on pig chromosome 4 used in the QTL analyses.

Marker name	Type of marker	References
*S0175*	Microsatellite	Ellegren & Basu 1995 [25]
*Sw839*	Microsatellite	Rohrer *et al*. 1994 [26]
*S0107*	Microsatellite	Ellegren *et al*. 1994 [27]
*Sw1089*	Microsatellite	Rohrer *et al*. 1994 [26]
*Sw1364*	Microsatellite	Rohrer *et al*. 1996 [28]
*RXRG*	SNP	Moller *et al*. 2004 [11]
*Sw714*	Microsatellite	Rohrer *et al*. 1996 [28]
*SDHC^a^/S0832*	Microsatellite	This study
*PEA15^a^/S0833*	Microsatellite	This study
*Sw1996*	Microsatellite	Rohrer *et al*. 1996 [28]
*Sw286*	Microsatellite	Rohrer *et al*. 1996 [28]
*S0214*	Microsatellite	Robic *et al*. 1995 [29]

^a^The marker was developed from a BAC containing a known gene, the gene name and the S number for porcine microsatellites are listed

### QTL analyses

**Figure 1:**
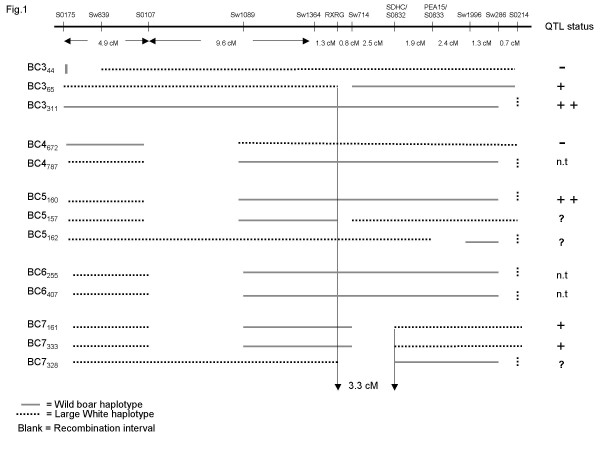
Summary of the genetic constitution as regards the *FAT1 *region of the backcross animals used for QTL analysis. The QTL status for each animal are presented; ++ = sire showing highly significant QTL effect; + = sire showing significant QTL effect; - = sire deduced to be not segregating for *FAT1*; ? = QTL data inconclusive; n.t. = not tested for QTL segregation. The refined *FAT1 *interval is indicated by vertical arrows and determined by the boars BC3_65_, BC7_161 _and BC7_333_, all segregating for the QTL. The map distances are from the linkage map by Moller *et al*. [11]. BCXy: BCX = backcross generation X, y = pig identity number.

**Figure 2:**
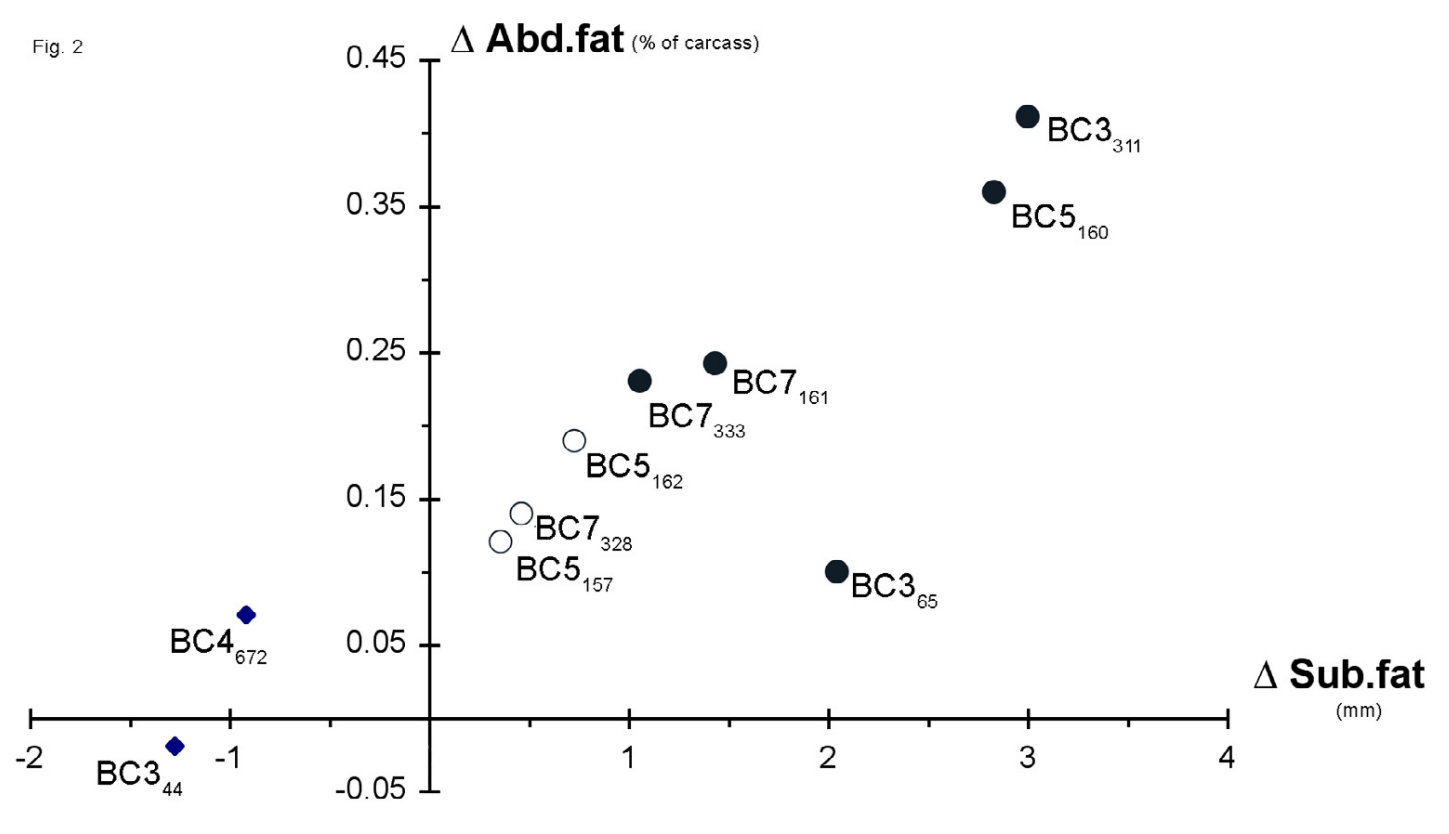
A graphic illustration of the estimated QTL effects on fatness traits for 10 backcross sires from a wild boar/Large White intercross. The *x*-axis represents Δ average subcutaneous fat and the *y*-axis represents Δ average abdominal fat (in both cases wild/domestic heterozygotes – domestic homozygotes). Boars represented by a black circle or a rectangle were deduced to be heterozygous or homozygous, respectively, at *FAT1*, whereas the QTL data were inconclusive for boars represented by a white circle. BCXy: BCX = backcross generation X, y = pig identity number.

The backcross generations and the results from the QTL analyses are summarized in Table 2 and in Figs. 1 and 2.

**Table 2:** Results from the analyses of the porcine *FAT1 *locus. The analyses are presented as least-square means (± standard errors) for different traits for each boar and genotype class. The number of records for each boar varies between phenotypic traits due to some missing values.

		Abdominal fat, % carcass			Subcutaneous fat depth (mm)			Lean meat + bone in ham, %			Sidefat. last rib (mm), ultrasound		
					
Boar	n^a^	w/d^b^	d/d^c^	*P*	w/d	d/d	*P*	w/d	d/d	*P*	w/d	d/d	*P*
44	6:53	1.78 ± 0.07	1.80 ± 0.08	0.83	19.3 ± 0.68	20.6 ± 0.80	0.23	77.0 ± 0.53	76.5 ± 0.62	0.55	16.0 ± 0.43	16.5 ± 0.48	0.44
65	6:56	1.82 ± 0.10	1.72 ± 0.10	0.49	22.2 ± 0.79	20.1 ± 0.70	0.07	75.4 ± 0.44	77.2 ± 0.39	0.005	17.8 ± 0.38	16.9 ± 0.36	0.11
311	7:63	1.93 ± 0.07	1.50 ± 0.08	0.000	19.5 ± 0.52	16.5 ± 0.56	0.000	76.5 ± 0.52	78.0 ± 0.57	0.07	16.5 ± 0.37	14.6 ± 0.41	0.001
672	6:46	1.75 ± 0.09	1.68 ± 0.08	0.56	15.1 ± 0.50	16.0 ± 0.49	0.18	77.6 ± 0.52	78.1 ± 0.49	0.49	14.0 ± 0.28	15.1 ± 0.27	0.003
160	6:46	1.78 ± 0.09	1.42 ± 0.09	0.01	17.1 ± 0.59	14.2 ± 0.56	0.002	79.2 ± 0.47	79.6 ± 0.45	0.59	15.7 ± 0.34	14.5 ± 0.34	0.01
157	6:53	1.69 ± 0.07	1.57 ± 0.06	0.20	15.7 ± 0.72	15.3 ± 0.63	0.70	79.0 ± 0.47	79.1 ± 0.41	0.87	14.0 ± 0.41	13.8 ± 0.38	0.79
162	6:43	1.86 ± 0.09	1.67 ± 0.09	0.14	16.0 ± 0.61	15.2 ± 0.57	0.40	79.3 ± 0.39	79.3 ± 0.37	0.90	14.4 ± 0.36	14.5 ± 0.34	0.81
161	7:56	1.35 ± 0.08	1.11 ± 0.06	0.03	17.4 ± 0.81	16.0 ± 0.61	0.16	77.0 ± 0.55	79.0 ± 0.42	0.01	11.4 ± 0.33	10.4 ± 0.27	0.03
333	6:55	1.71 ± 0.06	1.48 ± 0.06	0.01	18.8 ± 0.74	17.8 ± 0.71	0.31	76.0 ± 0.43	77.1 ± 0.41	0.08	12.6 ± 0.34	11.6 ± 0.34	0.05
328	7:49	1.48 ± 0.09	1.34 ± 0.08	0.27	14.8 ± 0.62	14.4 ± 0.54	0.59	79.3 ± 0.47	80.0 ± 0.41	0.28	10.1 ± 0.35	10.1 ± 0.31	0.99

^a^Number of litters:number of progeny

^b^Wild/domestic heterozygote

^c^Domestic homozygote

### QTL analyses in backcross 3 and backcross 4 boars

The QTL analysis of the backcross four (BC4) progeny showed that two of the BC3 boars (BC3_65 _and BC3_311_) were segregating for the *FAT1 *QTL, whereas BC3_44_, showed no indication of a QTL effect. For both BC3_65 _and BC3_311 _the wild boar haplotype was associated with higher fat deposition as expected. BC3_311 _was significant at the 1% level for abdominal fat, subcutaneous fat depth and for the ultrasonic side fat measurement. BC3_65 _was significant for the meat trait only, but showed a clear tendency for subcutaneous fat depth as well (P = 0.07). BC3_65 _harbors a smaller proportion of the wild chromosome and had less pronounced effects as compared to the BC3_311 _boar. Under the assumption that there is a single QTL and that both BC3_311 _and BC3_65 _are heterozygous for *FAT1*, the QTL interval was decreased to approximately 9.6 cM with *RXRG *and *S0214 *as flanking markers (Fig. 1).

BC4_672 _was selected to test for a possible additional QTL proximal to the wild/domestic breakpoint of BC3_65_. The result showed no QTL segregation for fatness/meat content traits in this interval. BC4_672 _was significant for side fat at the last rib but with an opposite trend, the domestic homozygote having higher fat deposition (Table 2). We conclude that BC4_672 _did not carry the wild boar allele for the *FAT1 *QTL. Thus, we can exclude the region proximal to marker *S0107 *as associated with the *FAT1 *QTL (Fig. 1).

### QTL analyses in backcross 5 boars

Sow BC4_787 _gave birth to 10 offspring. Two recombinant boars and one boar carrying the same haplotype as 787 were selected for QTL analysis. The progeny testing from the BC5 boars showed that the *FAT1 *QTL was clearly segregating in boar BC5_160 _which carried the same haplotype as its mother (BC4_787_). The BC5_160 _boar was highly significant for the same phenotypic measures as the BC3_311 _boar. The QTL analysis for the other two recombinants (BC5_157 _and BC5_162_) were considered inconclusive since there was a tendency for a QTL effect (the wild boar haplotype associated with higher fat deposition) but it did not reach statistical significance for any trait (Table 2, Fig. 2).

### QTL analyses in backcross 7 boars

Two sons from BC5_160 _were selected for further breeding. These two boars, BC6_255 _and BC6_407_, generated three interesting recombinants out of a total of 395 offspring; BC7_161 _from BC6_255 _and the siblings BC7_328 _and BC7_333 _from BC6_407_. The *FAT1 *QTL was concluded to be heterozygous in two of these three boars: BC7_161 _and BC7_333 _(Table 2, Fig. 2). Both these boars were significant for abdominal fat and side fat at the last rib. BC7_161 _was also highly significant for lean meat content. None of them were however significant for subcutaneous fat depth but the expected trend of higher subcutaneous fat associated with the wild boar allele was present. The data for BC7_328 _were inconclusive since it showed a non-significant trend for the wild boar haplotype to be associated with higher fat deposition.

### Definition of the *FAT1 *interval

Based on the data presented in this study and under the assumption that there is a single underlying locus for *FAT1 *we can reduce the critical interval to only 3.3 cM with *RXRG *and *SDHC/S0832 *as flanking markers. This is the only shared chromosome fragment among the five sires that showed significant QTL effects (Fig. 1). However, at present we cannot exclude the possibility that more than one gene is underlying this QTL and if this is the case the critical region is still broad (see Discussion).

## Discussion

In this study we have been able to follow the segregation of the *FAT1 *QTL over six generations of marker-assisted backcrossing. As a result the localization of this major QTL has been refined considerably. Positional cloning of QTLs are challenging for several reasons particularly for outbred species [16]. In our study we have made backcrossing to Large White sows with the assumption that this breed is fixed for a QTL allele associated with low fat deposition due to the very strong selection for lean growth in this breed. However, we cannot excluded the possibility that the wild type allele remains segregating at a low frequency in the domestic line which implies that the lack of QTL segregation may sometimes occur because a backcross sire is homozygous for the wild type-allele at *FAT1*. Thus, haplotype data obtained from segregating sires should be given more weight than haplotype data from non-segregating sires. A second complication may occur since we do not know if the large effect associated with *FAT1 *is due to a single gene or two (or more) linked genes on chromosome 4. In the latter case, the *FAT1 *locus will break up into multiple QTLs with minor effects during the course of introgression. This study was designed to distinguish between segregation at a QTL with major effects on fatness versus no QTL segregation, but the sizes of the progeny groups have not been sufficiently large to reliably resolve a more complicated genetic architecture. Finally, the QTL effects may change as the wild type allele at *FAT1 *is introgressed on another genetic background due to epistatic interaction. It is well established that epistatic interactions may contribute significantly to the genetic basis for multifactorial traits [17].

We have investigated the QTL status of 10 backcross sires and concluded that five were segregating for the QTL, two were negative while the data were inconclusive for the remaining three (Fig. 2). The estimated QTL effects for two sires (BC3_311 _and BC5_160_) were very similar to those estimated using the F_2 _generation [1]. Based on the genetic composition of these two sires we can therefore conclude that the mutation or mutations underlying the major effects associated with the *FAT1 *locus is located in the 20 cM interval between markers *S0107 *and *S0214*. The estimated effects for the three other sires showing QTL segregation (BC3_65_, BC7_161 _and BC7_333_) were markedly lower but the statistical analysis did not reveal a significant genetic heterogeneity in QTL effects among the five sires. Thus, we cannot exclude the possibility that they have the same QTL genotype and that the variation in QTL effects are due to random sampling. Under the assumption that these five sires are heterozygous for the same QTL mutation(s) we can reduce the critical interval for *FAT1 *to the 3.3 cM interval between the flanking markers RXRG and SDHC/S0832 (Fig. 1). However, our data are also consistent with a model in which *FAT1 *reflects the segregation at two different loci in the 20 cM interval between *S0107 *and *S0214*. Under this scenario BC3_311 _and BC5_160 _should be segregating at both loci whereas BC7_161 _and BC7_333 _should only be segregating for a proximal locus located in the interval *S0107-SDHC/S0832 *and BC3_65 _should be heterozygous for a more distal locus in the interval (*RXRG-S0214*). This two-locus model gains some support from the fact that the two sires carrying the largest haplotype block from the wild boar also showed the largest QTL effects.

In the BC2 generation the QTL effect for both growth and fat deposition was confirmed [10]. In this study we have been able to confirm QTL effects on fatness but we found no evidence for QTL effects on growth traits including birth weight, daily weight gain and length of carcass (data not shown). We conclude therefore that there must be different QTLs on chromosome 4 controlling fatness and growth.

Pérez-Enciso *et al*. [6] identified a major QTL affecting fatness on pig chromosome 4 using an Iberian/Landrace intercross and the location and QTL effects were strikingly similar to our data from the wild boar/Large White intercross. In a recent study Mercadé *et al*. [5] have preformed a multitrait, multi-QTL analysis in order to deduce if there are more than one QTL on SSC4 and to refine the position of the QTL. They found indications of two QTL influencing body composition. The most significant one has a large effect on fatness and maps close to the *FABP4 *gene at 70 cM; *FABP4 *encodes adipocyte fatty-acid binding protein and is thus a potential candidate gene for the *FAT1 *locus. However, we can exclude *FABP4 *as underlying the major QTL for fatness in our wild boar/Large White intercross since this gene is located proximal to the recombination break-point carried by sire BC5_160 _(Fig. 1). The second QTL proposed by Mercadé *et al*. [5] has an affect on growth and is located at about 90–95 cM, in the interval between marker *S0073 *and *S0214*. The location of our *FAT1 *QTL overlaps the one for this second QTL influencing growth, however we did not reveal any QTL effects on growth traits in the present study. Thus, it appears to be some significant differences in QTL compositions between the two pedigrees.

The 3.3 cM region shared by all segregating backcross sires (Fig. 1) is orthologous to a region at HSA1q22-24 with very high conservation of gene order between the two species [11]. The flanking markers *RXRG *and *SDHC *refine the position on the human map to 1q23.3 according to the data presented by Moller *et al*. [11]. The relationship between the porcine RH map (Ray) and the physical distance (Mbp) in human is almost linear in this region and has been estimated to 3.5 Mbp/Ray across the HSA1q arm. The RH map position in Ray for *RXRG *and *SDHC *are 22.8 and 23.5, respectively. This suggests that our QTL interval is approximately 2.5 Mbp. The orthologous region in the human genome harbors two interesting candidate genes for *FAT1*, LIM homeobox transcription factor 1 alpha (*LMX1A*) and pre-B-cell leukemia transcription factor 1 (*PBX1*). *LMX1A *encodes a transcription factor expressed in pancreas that has been shown to activate insulin gene transcription [18]. *PBX1 *is essential for normal pancreatic development and function [19,20] and has shown a modest association to Type 2 diabetes susceptibility in humans [21].

The minimum interval of 3.3 cM for *FAT1*, assuming the single-gene model, covers a region which is too large to sequence and too small to perform further backcrossing in order to try to generate new recombinants. However, several of the segregating sires (BC3_65_, BC5_157_, BC7_161 _and BC7_333_) carry haplotypes that are recombinant between the flanking markers of the QTL interval (Fig. 1). By identifying more markers and type these markers in the recombinant pigs we will be able to decrease the interval further. Another approach to consider is Identity-By-Descent (IBD) mapping. In a recent study Van Laere *et al*. [22] used this approach when they identified a quantitative trait nucleotide underlying a major QTL influencing muscle growth, fat deposition, and heart size in the pig. The IBD approach could be applied in our study since we believe that one or more favorable mutations reducing fat deposition have gone through a selective sweep in domestic lines. Many domestic lines have been intensively selected for growth and lean meat, like the Large White line, and may thus share a haplotype that are IBD and that carry the causative mutation(s) for *FAT1*.

## Conclusion

This study is a continuation of the work published by Marklund *et al*. [10] where the *FAT1 *QTL was confirmed in a backcross population and the QTL interval was defined to be as large as 70 cM. We have now refined the localization of the *FAT1 *QTL on pig chromosome 4 by marker assisted backcrossing for six additional generations of our Large White/wild boar intercross. The region harboring *FAT1 *is now reduced to ~20 cM if we allow for the possibility of multiple genes underlying this QTL whereas the critical interval becomes as small as 3.3 cM if we assume that *FAT1 *represents a single gene effect (Fig. 2). The flanking markers of the latter interval are *RXRG *and *SDHC*. The orthologous region of *FAT1 *in the human genome is located on HSA1q23.3 and harbors approximately 20 genes with *LMX1A *and *PBX1 *being the most interesting positional candidate genes.

## Methods

### Animals and the backcross procedure

The backcross boars used in this study belong to a multigeneration pedigree originating from an intercross between two European wild boars and eight domestic Large White sows [1]. The *FAT1 *locus originally identified using the F_2 _generation [1] was subsequently confirmed in a backcross pedigree, generated from two selected recombinant boars, and comprising a total of 85 animals [10]. Following these initial studies we have traced the inheritance of this QTL through another six backcross generations.

In each generation, new boars carrying a smaller and smaller proportion of wild boar-derived segments of chromosome 4 have been selected for breeding using marker assisted selection (Fig. 1). The selected boars were backcrossed to Large White sows and at least 50 progeny from each recombinant boar were generated in order to give sufficient statistical power to judge whether the boar was segregating for the *FAT1 *QTL or not.

Three recombinant boars, denoted BC3_65_, BC3_44 _and BC3_311_, were generated from backcross generation 3 (BC3). Following QTL analysis, two recombinant animals was selected from offspring to BC3_311_; one boar (BC4_672_) and one sow (BC4_787_) being heterozygous wild/domestic for different parts of chromosome 4 (Fig. 1). Since there were no boars with recombinant haplotypes among the BC3_311 _offspring, we had to select a sow to generate new boars for the next backcross generation. Out of 10 offspring from sow BC4_787 _two recombinant boars were selected, BC5_157 _and BC5_162_, and one boar, BC5_160_, carrying the same haplotype as the sow. Progeny testing was performed and two boars, BC6_407 _and BC6_255_, were selected and 137 and 258 offspring were produced, respectively, in order to identify new recombinants. From BC7 three recombinant boars were selected (BC7_161_, BC7_328 _and BC7_333_) and used for QTL analyses (Fig. 1).

All animals were reared at the Swedish University of Agricultural Sciences pig research station at Funbo-Lövsta. Animals were weaned at five weeks of age and males were kept intact. At nine weeks of age the animals were sorted by sex and weight and put into groups of eight. The pigs were fed a standard diet with on average 12.2 MJ and 16% cp. Slaughter was performed at approximately 100 kg.

### Phenotypic measurements

Phenotypic measurements were collected from all animals. Back fat thickness was measured at the last rib on live animals using ultrasound scanning at a weight of approximately 90 kg. After slaughter subcutaneous fat depth at the last rib was measured on the carcass. Flares were weighted and percentage abdominal fat was calculated in the carcass. The carcass was then partially dissected and the percentage meat and bone in ham was calculated. The phenotypic traits analyzed as well as the number of records for each trait are presented in Table 2.

### Genetic markers

All genetic markers used in this study, except *SDHC/S0832 *and *PEA15/S0833*, have been described previously (Table 1). The *SDHC*/S0832 and *PEA15*/S0833 microsatellites were isolated as follows; the porcine BAC library RPCI44 [23] was screened with gene specific probes for *SDHC *and *PEA15*. Two positive BAC clones, BAC RPCI44-310B8, containing *SDHC*, and BAC RPCI44-391C14, containing *PEA15*, were isolated and subsequently screened for microsatellites as previously described [24]. The primer sequences for microsatellite *SDHC *are; forward 5'-CGCACTGGGAACTCCATATGC-3' and reverse 5'-TTTTATTCTAGCAGTTGTTTCCCCC-3', and for PEA15; forward 5'-CACACCCATGCATTCACACCAG-3' and reverse 5'AGGAACATGGGCTCAGCCAAG-3'. The microsatellites were amplified using a touchdown PCR profile described in Moller *et al*.  [11] with 50 ng genomic DNA in a total volume of 10 μl. PCR amplified microsatellites were analyzed with capillary electrophoresis using MegaBASE 1000 sequencer and the Genetic profiler software version 2.2 (Amersham Biosciences, Uppsala, Sweden).

### QTL analysis

The data were analyzed using Proc GLM in the SAS-package version 9.1 [SAS Inst., Inc., Cary, NC]. Each sire family was analyzed separately. The model included the effect of dam, sex and marker. Dam, sex and genotype were treated as fixed effects in the analysis. Carcass weight was included in the model when analyzing subcutaneous fat depth. For the QTL analyses, the BC progeny were classified as wild/domestic heterozygotes and domestic homozygotes using genetic markers and with reference to the specific chromosome segment for which the sire was heterozygous (wild/domestic). The QTL analysis was, for each boar, carried out on this classification and not on each individual marker. Consequently, all recombinant offspring were excluded in the QTL analysis.

## Authors' contributions

FBcarried out DNA extraction and genotyping on the BC6 and BC7 progeny, summarized all data for the backcross generations and prepared the manuscript. SSperformed parts of the statistical analysis, managed the pigs and collected and put together all phenotypic data. KA performed most of the statistical analysis.

LA conceived and supervised this study, edited the manuscript and made the final improvements of the manuscript. MMcarried out DNA extraction and genotyping of the BC3, BC4 and BC5 progeny, cosupervised the study and edited the manuscript. All authors read and approved the final manuscript.
